# Tailoring Charged Nanofiltration Membrane Based on Non-Aromatic Tris(3-aminopropyl)amine for Effective Water Softening

**DOI:** 10.3390/membranes10100251

**Published:** 2020-09-24

**Authors:** Pengrui Jin, Michiel Robeyn, Junfeng Zheng, Shushan Yuan, Bart Van der Bruggen

**Affiliations:** 1 Department of Chemical Engineering, KU Leuven, Celestijnenlaan 200F, B-3001 Heverlee, Belgium; pengrui.jin@kuleuven.be (P.J.); michiel.robeyn@student.kuleuven.be (M.R.); junfeng.zheng@kuleuven.be (J.Z.); 2 School of Environmental Science & Engineering, Huazhong University of Science and Technology, Wuhan 430074, China; 3 Faculty of Engineering and the Built Environment, Tshwane University of Technology, Private Bag X680, Pretoria 0001, South Africa

**Keywords:** tris(3-aminopropyl)amine, nanofiltration membrane, positively charged membrane, water softening

## Abstract

High-performance positively-charged nanofiltration (NF) membranes have a profound significance for water softening. In this work, a novel monomer, tris(3-aminopropyl)amine (TAEA), with one tertiary amine group and three primary amine groups, was blended with trace amounts of piperazine (PIP) in aqueous solution to fabricate a positively-charged NF membrane with tunable performance. As the molecular structures of TAEA and PIP are totally different, the chemical composition and structure of the polyamine selective layer could be tailored via varying the PIP content. The resulting optimal membrane exhibited an excellent water permeability of 10.2 LMH bar^−1^ and a high rejection of MgCl_2_ (92.4%), due to the incorporation of TAEA/PIP. In addition, this TAEA NF membrane has a superior long-term stability. Thus, this work provides a facile way to prepare a positively charged membrane with an efficient water softening ability.

## 1. Introduction

With the emergence of population explosion, industrialization, and water pollution, the water crisis has become one of the most threatening global challenges [1,2,3]. Water treatment will be an integral part of solving water scarcity, and membrane separation technologies will be indispensable in this effort, thanks to their advantages of low energy inputs, high efficiency, and easy scale-up over conventional treatment methods [4]. Nanofiltration (NF) membranes with pore sizes of 0.5–2 nm can readily separate multivalent ions in hard water (mainly containing high concentrations of magnesium (Mg^2+^) and calcium ions (Ca^2+^)) in view of water softening [5,6]. Thus, this promising membrane system can solve various problems related to scaling in both industrial and residential settings, such as clogging of water tubes and deterioration of equipment [6,7].

Currently, typical NFs based on thin-film-composite (TFC) polyamide (PA) membranes are formed by interfacial polymerization (IP) between amine monomers (in aqueous solution) and acyl chloride monomers (in organic solvent) on an ultrafiltration support [8,9,10]. The excess acyl chloride groups can be hydrolyzed into carboxyl groups in the aqueous environment, causing the PA selective layers to be negatively charged, which then exhibit a low rejection to multivalent cations and are ineffective for water softening [11,12,13]. Therefore, the development of positively charged NF membranes continues to attract research interest, due to the use of positively charged NF membranes for water softening allowing the effective repelling of multivalent cations [14,15,16]. Conventional positively charged NF membranes are able to effectively separate cations thanks to the main rejection mechanisms of size sieving [17], electrostatic repulsion (the Donnan effect) [18], and the dielectric effect [19,20]. While recent studies have explored several methods (such as IP [21,22], surface coating [6,23,24], layer-by-layer [25], and phase separation [26]) to develop positively charged NF membranes with a high water permeance and a good salt rejection, simple and effective methods of IP based on new materials for preparing positively charged NF membranes are worth exploring.

Tris(3-aminoethyl)amine (TAEA), a novel tertiary amine with three primary amine groups and a small molecular size, is a promising monomer for preparing TFC PA NF membranes with positive charges. In the IP process, TAEA can increase the positive surface charges in multiple ways. TAEA with tertiary amine groups are easy protonated on the PA layer, leading to a positively-charged membrane surface [27]. Meanwhile, TAEA with tertiary amino groups can adsorb the by-product hydrogen chloride of the IP reaction, accelerating the reaction between amine groups and acyl groups, thereby minimizing the residual carboxyl groups and reducing the negative charge [28]. However, to the best of our knowledge, no study has yet been reported on the fabrication of TFC NF membranes in which TAEA plays this dual role.

It has been widely accepted that electrostatic repulsion, the dielectric effect, and size exclusion determines the rejection mechanisms of NF membranes [17,18,29]. Therefore, research about pore structures and surface charges of NF membranes is important for developing high performance membranes for water softening. Hence, in this study, a number of positively charged NF membranes were first prepared by adding piperazine (PIP, to adjust pore structure and surface charges) into TAEA solution via IP with trimesoyl chloride (TMC), and the effects of different contents of PIP on the physicochemical properties and performance of the resulting membranes were comprehensively investigated. The prepared membranes were extensively characterized to confirm the successful formation of various PA layers and to evaluate their effect on NF properties. Moreover, the IP time of the NF membrane was also systematically optimized. Finally, the resultant optimal NF membrane has a high perm-selectivity as well as a long-term stability for water softening.

## 2. Materials and Methods

### 2.1. Chemicals and Materials

Polyethersulfone (PES) (Veradel 3000P) was purchased from BASF Co. (Oudenaarde, Belgium). Dimethyl sulfoxide (DMSO, 99.5%) from Sigma-Aldrich (Diegem, Belgium) was used as solvent for the dope solution, and deionized water was used as the solvent and coagulant for ultrafiltration (UF) membrane fabrication. Tris(3-aminoethyl)amine (TAEA, ≥97%), piperazine (PIP, 99%), trimesoyl chloride (TMC, 98%), and n-hexane (95%) purchased from Sigma-Aldrich (Diegem, Belgium) were used to prepare PA TFC membranes. Various salts including magnesium chloride (MgCl_2_), magnesium sulfate (MgSO_4_, 99%), sodium chloride (NaCl, 99%), and sodium sulfate (Na_2_SO_4_, 99%) from Sigma-Aldrich (Diegem, Belgium) were used to test salt rejection at an inlet concentration of 1.0 g L^−1^. Unless specified, the aqueous solutions were prepared by deionized water purified through a Milli-Q ultrapure unit (Darmstadt, Germany).

### 2.2. Fabrication of TFC NF Membranes

**Figure 1:**
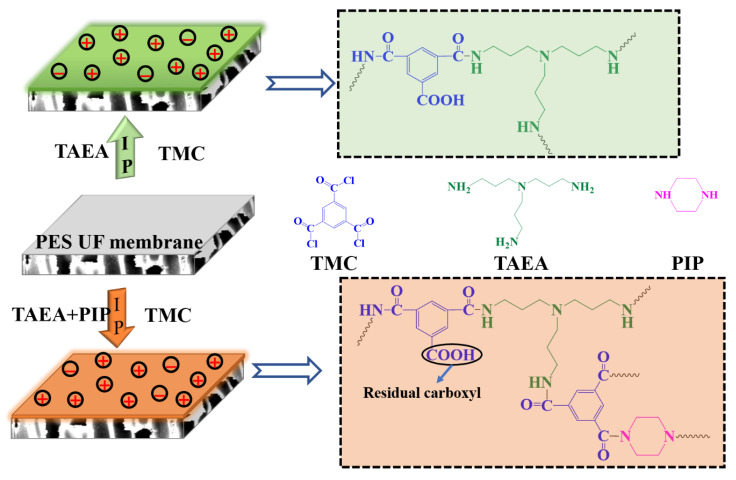
Schematic diagram of thin-film-composite nanofiltration (TFC NF) membranes fabrication.

The TFC NF membranes were fabricated via the IP method and the process is presented in Figure 1. The PES UF membranes were prepared by the non-solvent (water) induced phase separation method via casting the dope solution with a composition of PES/DMSO (15/85 wt%) on a non-woven fabric using a casting knife with a thickness of 250 um, more details can be found in our previous work [30].

The PES substrates (water permeance, around 1200 LMH bar^−1^) were firstly immersed into a TAEA/PIP aqueous amine solution for 5 min, where the TAEA concentration was fixed at 0.08 wt%, while the PIP concentration was varied in the range of 0–0.04 wt% for adjusting pore structures and surface charges. Afterwards, the aqueous solution was removed from the holder, and the residual water on the PES membrane surface was gently removed with an air knife. Then the TMC solution was poured onto the membrane surface for 1 min at a constant temperature of 25 °C ± 3 °C and a relative humidity of 33% ± 3% which resulted in the formation of a PA active layer over the PES substrate. The resulting NF membrane was rinsed with hexane to remove unreacted TMC, then stored in deionized water at 4 °C before use. The denotation ‘TAEA-PIPx’ represents a TAEA-PIP membrane prepared from an aqueous solution with PIP concentration of x%.

### 2.3. Characterization Methods

The surface elemental composition of PA active layers was analyzed using a NEXUS670 Fourier transform infrared (FTIR) spectrometer and a Krais ULFRA X-ray photoelectron spectroscope. SEM images were obtained using a Philips field emission scanning electron microscopy at an accelerating voltage of 10 kV. Cross-section samples were prepared by freeze fracturing the membranes in liquid nitrogen. Samples were sputtered with a thin layer of gold (Au) to reduce charging during imaging. The hydrophilicity of NF membranes was evaluated using an OCA20 instrument system with 2 µL water droplets at 5 random locations. The surface streaming potential of the membrane surface was investigated using a zeta-potential and particle size analyzer mastersizer NaNoZs.

### 2.4. Membrane Performance Evaluation

The NF performance was evaluated in a lab made cross-flow filtration cell with an active membrane area of 22.9 cm^2^ [31]. Prior to filtration, all membranes were pre-pressurized with DI water for at least 30 min at 6 bar util a steady water flux was attained. After compaction, the water flux was measured for each membrane at 4 bar and room temperature with a flow velocity of 40 L h^−1^. The average water flux (J, L m^−2^ h^−1^, or LMH) was calculated as follows: *J* = *V*/(*A*·Δ*t*), where *V* represents the volume of permeated liquid (L) collected over a period of time Δ*t* (h), and *A* is the effective membrane area (m^2^). The pure water permeance (PWP, L m^−2^ h^−1^ bar^−1^) was calculated by the following equation: *PWP* = *J*/Δ*p*, where Δ*p* is the applied pressure.

The feed concentration of salts (MgCl_2_, MgSO_4_, NaCl, Na_2_SO_4_) was 1000 ppm, and salt rejection was calculated based on the electrical conductivity of the feed and permeate under a stable permeating flux. Rejection of PEG molecules (200, 400, 600, and 800 Da, 200 ppm) was also evaluated by measuring the concentration of the feed and permeate solutions using a UV-visible absorption spectrophotometer at 535 nm with BaCl_2_ and I_2_/KI solutions as chromogenic reagents to determine the molecular weight cutoff (MWCO) of the NF membranes [29,32]. The retention of salts and organic species was separately tested at 4 bar and room temperature. The rejection was calculated as *R* = (1 − *C*/*C_f_*) × 100%, where *C_p_* and *C_f_* (g L^−1^) are the solute concentrations in the feed and permeate solution, respectively. All the PEG concentrations were measured with a Shimadzu UV-spectrophotometer. The single salt concentrations in the feed and permeate were measured by a Thermo Scientific Orion Star A212 conductivity meter.

All measurements including water permeance and solute rejection were recorded from three parallel experiments.

## 3. Results and Discussion

### 3.1. Characterizations of the Prepared Membranes

**Figure 2:**
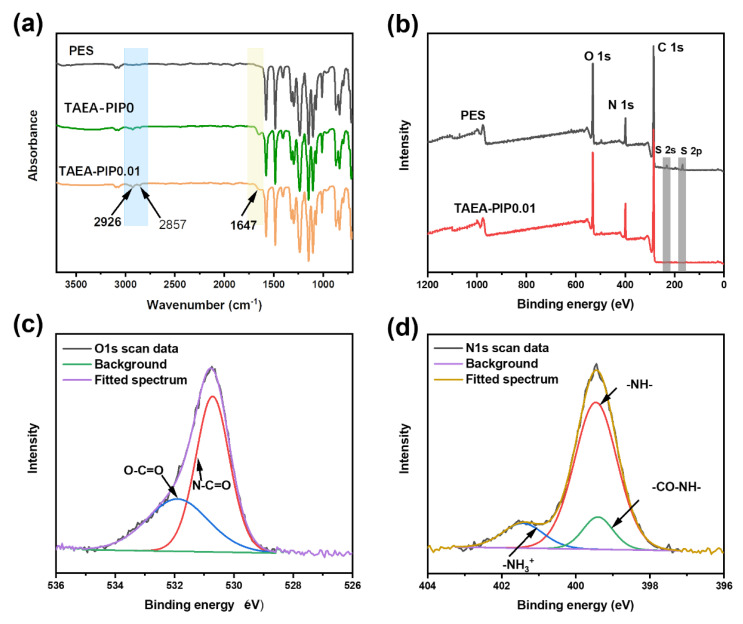
(**a**) FTIR spectra of the polyethersulfone (PES), tris(3-aminopropyl)amine (TAEA)-PIP0, and TAEA-PIP0.01 membranes; (**b**) XPS survey spectra of the PES and TAEA-PIP0.01 membranes; High-resolution XPS spectra of (**c**) O1s and (**d**) N1s for the TAEA-PIP0.01 membrane.

To confirm the presence of a PA layer on a PES support membrane, the chemical composition of the obtained membrane surface was investigated by XPS and FTIR analysis. Figure 2a presents the FTIR spectra of the PES substrate and the TFC PA membranes. Compared with the PES support membrane, the obvious adsorption signal found in TAEA-PIP0 and TAEA-PIP0.01 around 1647 cm^−1^ was ascribed to C=O stretching (amide I), and a very weak peak around 1540 cm^−1^ was ascribed to –NH of amine II, implying the formation of the amide group [33,34]. Meanwhile, the peaks at 2936 cm^−1^ and 2857 cm^−1^ were attributed to the –CH_2_– group derived from the TAEA chain. The results clearly demonstrated that the PA layers were formed. The XPS spectrum (Figure 2b) displayed that the emission peaks of S 2s (233.3 eV) and S 2p (167.8 eV) derived from the PES substrate substantially weakened for the TAEA-PIP0.01 membrane, further confirming the presence of PA layers on the substrate. The deconvolution of O 1S spectra (Figure 2c) of TAEA-PIP0.01 membrane involves two peaks: N–C=O at 530.9 eV and O−C=O at 532.1 eV [35,36]. The N1s peak (Figure 2d) of the TAEA-PIP0.01 membrane can be deconvoluted into three peaks, i.e., –NH– at 399.7 eV, –CO–NH– at 399.5 eV, and NH_3_^+^ at 401.7 eV [37,38]. The spectrum of O1s and N1s proved that TAEA/PIP and TMC could be amidated under the IP process. The majority of the structure of PA active layers was formed by amidation, but there were still carboxyl groups formed by hydrolysis of acid chloride on TMC and protonated amino groups [11,22].

**Figure 3:**
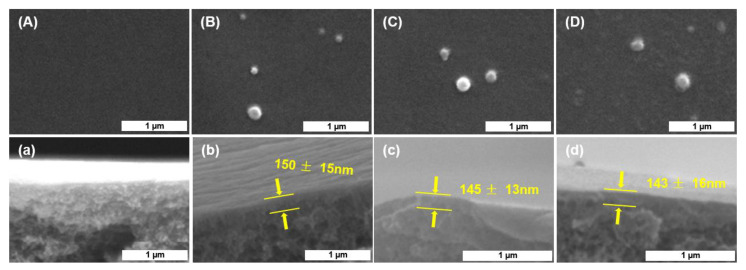
The surface morphology of (**A**) PES, (**B**) TAEA-PIP0, (**C**) TAEA-PIP0.01, (**D**) TAEA-PIP0.02 membranes; Cross-sectional morphology of (**a**) PES, (**b**) TAEA, (**c**) TAEA-PIP0.01, (**d**) TAEA-PIP0.02 membranes.

Surface and cross-section SEM images of the PES and TFC NF membranes are shown in Figure 3. The prepared PES membranes (Figure 3A,a) exhibited a typical asymmetric structure, including a finger-liker sublayer and a thin-skin layer with small surface pores [39]. Figure 3 proves that the PA active layer was steadily polymerized on the surface of the PES support without apparent defects. Furthermore, the sporadic protrusions on the surface of the TFC NF membranes (Figure 3B–D) were formed during the IP processes [10]. From the cross-section morphology of the TFC NF membranes (Figure 3a–d), it can be observed that the thickness of all the PA active layers was around 145 nm and did not change greatly with the monomer concentration. The increase in PIP concentration could have promoted the monomer diffusion rate to increase the thickness of polyamide layer [40], while the intensification of the self-limiting reaction caused by the addition of PIP could have limited the increase in the thickness of the polyamide layer [41]. The mutual restriction of the two resulted in no significant change in the thickness of the polyamide layer.

### 3.2. Optimization of Separation Performance

**Figure 4:**
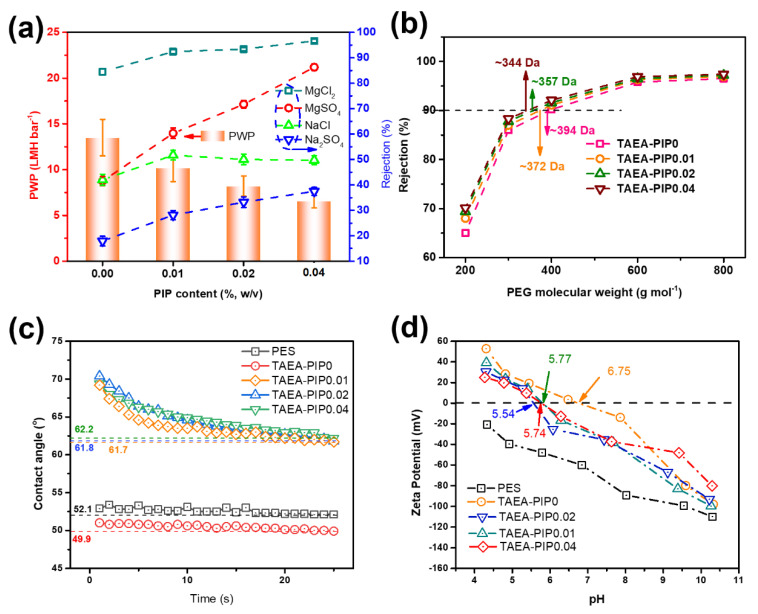
(**a**) Water permeance and salt rejection of membranes with different concentrations of PIP monomer; (**b**) Zeta potential and (**c**) dynamic water contact angles of PES, TAEA-PIP0, TAEA-PIP0.01, TAEA-PIP0.02, and TAEA-PIP0.04 membranes; (**d**) MWCOs of TAEA, TAEA-PIP0.01, TAEA-PIP0.02, and TAEA-PIP0.04 membranes tested with 200 mg L^−1^ PEG aqueous solution at 4 bar.

The separation performance of TAEA-PIP membranes was optimized by varying the PIP concentration. Figure 4a gives the single salt rejection (MgCl_2_, MgSO_4_, NaCl, Na_2_SO_4_) at 1 g/L and the water permeance of TFC PA NF membranes prepared under different PIP concentrations. The pH values of the four feed solutions were 7.50, 6.77, 6.59, and 6.22, respectively. For the different concentrations of PIP monomer, the water permeance diminished from 13.5 to 6.6 LMH bar^−1^ by enhancing the PIP concentration from 0.00 to 0.04 w/v%. According to the definition of the MWCO, pore structure can be estimated in terms of MWCO [21,29,42]. As confirmed by the MWCO analysis (Figure 4b), the MWCO of the TFC NF membranes decreased with the enhancement of the PIP concentration in the aqueous phase. Thus, TAEA-PIP0.04 had the smallest pore size; and therefore it should have the densest PA layer and low water permeance. This decrease in the MWCO caused by the high PIP concentration resulted in a decrease of the water permeance. The water contact angles (WCAs) of the TAEA-based membranes were investigated (Figure 4c), and were found to follow the sequence: TAEA-PIP0 (49.9°) < TAEA-PIP0.01 (61.8°) < TAEA-PIP0.02 (62.2°) < TAEA-PIP0.04 (62.5°). It could be observed that the WCA of the TFC membranes increased considerably with increasing PIP concentration, which also led to the decrease in water permeance of the TAEA-PIP membranes. All of the TAEA-PIP NF membranes exhibited a high selectivity for four salts (following the order MgCl_2_ > MgSO_4_ > NaCl > Na_2_SO_4_). This trend indicates the typical characteristic of positively charged TFC NF membranes [22]. Compared with MgCl_2_, the membrane is less repellent to MgSO4. This is due to the presence of divalent SO_4_^2^ ions, which greatly affects the cationic electric field provided on the membrane surface and reduces the electrostatic repulsion effect [43]. When the concentration of PIP was 0.00%, the water permeance of the TAEA-PIP0 NF membrane was high, but the salt rejection was not sufficiently high. Thus, PIP was chosen to modify the PA cross-linked network. The rejection of MgCl_2_ increased from 84.5% to 96.6, and the rejection of MgSO_4_ increased from 41.5% to 86.3%, as the concentration of PIP increased from 0.00% to 0.04%. The increased rejection of MgCl_2_ and MgSO_4_ was mainly caused by the dielectric exclusion and the decreased pore size, as confirmed by the MWCO analysis (Figure 4b). The salt rejection of NF membranes is mainly governed by size exclusion, dielectric exclusion, and electrostatic repulsion mechanisms [44]. The increasing of PIP concentration leads to a change in the ratio of amine and carboxylic acid groups at each axial position along the pore length, this asymmetric charge property contributes better charge solute rejection performance [19,45]. To better understand the membrane rejection characteristics, the surface charge of the PES substrate and the TAEA-PIP membranes with various concentrations of PIP were further studied by measuring zeta potentials. As shown in Figure 4d, the electro-positivity of the TAEA-PIP0 membrane was stronger than that of the TAEA-PIPx membranes, which was mainly due to the TAEA with protonated amine groups and less carboxyl groups, and with negative charge on the PA layer. As a result, the TAEA-PIP0 membrane should have had the highest MgCl_2_ rejection, but the observations were completely the opposite. Thus, the salt rejection behavior of the resulting TAEA-PIP membranes depended on size-based sieving when the electro-positivity was similar [10,46]. Taking into account both water permeance and salt rejection, the TAEA-PIP0.01 NF membrane was selected for further studies. Furthermore, with the increasing of PIP concentration in aqueous phase, the isoelectric point of the membranes became lower (Figure 4d). In the case of a certain amount of TMC, more PIP involved in the IP reaction could reduce the protonated amine groups from TAEA in the formed polyamide layer, leading to the decrease in isoelectric point.

**Figure 5:**
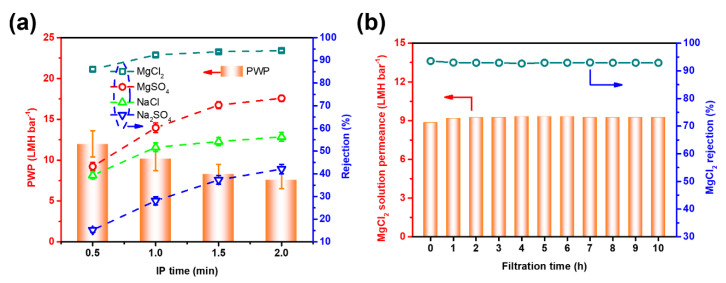
(**a**) The effect of IP time on NF performance of TAEA-PIP NF membranes, keeping the concentration of 0.01% PIP constant; (**b**) long-term stability of the prepared TAEA-PIP0.01 NF membrane, 1 g/L MgCl_2_. (4bar, 25 ± 3 °C).

In order to optimize the membrane performance, the IP reaction time was evaluated based on the membrane preparation conditions of TAEA-PIP0.01 with various reaction times. As shown in Figure 5a, the water permeance declined from 12.0 to 7.6 LMH bar^−1^ when the IP reaction time increased from 0.5 to 2 min. Conversely, the salt rejection increased with extended IP reaction time from 0.5 to 2 min. For example, the MgCl_2_ rejection increased from 86.1% to 94.3%. With increasing IP reaction time, the PA active layer became thicker and the degree of cross-linking also improved, thereby reducing the water permeance and salt rejection [47]. Due to a self-limiting phenomenon in the IP process, the water permeance and the salt rejection are nearly constant after a certain period of IP time [48]. Taking into account both water permeance and salt rejection, the optimal reaction time was designated as 1 min in this study.

### 3.3. Stability of TAEA-PIP0.01 NF Membrane and Performance Comparison

Figure 5b shows the performance of the TAEA-PIP0.01 membrane for purifying a MgCl_2_ solution for 10 h continuous filtration at 4 bar. During 2 h, the solution permeance increased slightly, which was due to the influence of continuous filtration leading to slight loosening of the partial ion crosslinking between TAEA and TMC [22]. After that, the permeance and rejection were constant. Thus, the TAEA-PIP0.01 membrane was found to have an extraordinary stability for filtration of a saline solution. Table 1 summarizes a comparison of the separation performance of the TAEA-PIP0.01 membrane and the other NF membranes. It can be seen that TAEA-PIP0.01 has a competitive performance. Compared with NF membranes reported in the literature, the membrane in this work was found to have a comparable water permeance (10.2 LMH bar^−1^) and MgCl_2_ (92.4%) rejection, indicating its outstanding performance for water softening. In addition, comparing the rejection of NaCl, it can be found that the membrane prepared in this work is relatively dense.

## 4. Conclusions

In this study, a novel positively charged NF membrane was developed via IP reaction on a PES substrate, with TMC and TAEA/PIP serving as the organic and aqueous phase monomers, respectively. The addition of PIP into the aqueous phase during IP reaction between TAEA and TMC led to an adjustment of the pore size and chemical properties of the PA film, thereby improving the NF performance. When the PIP content was 0.01 w/v%, the optimized TAEA-PIP0.01 membrane yielded a remarkable water permeance (10.2 LMH bar^−1^) and salt rejection (MgCl_2_, 92.4%). In addition, the resultant membrane had an excellent stability. This study provides an effective method to tailor NF membranes with tunable separation performance for water softening.

## Figures and Tables

**Table 1:** Comparison of TAEA-PIP0.01 NF membrane with state-of-the art works.

Membrane	PWP (LMH bar^−1^)	Conditions	MgCl_2_ Rejection (%)	NaCl Rejection (%)	Ref.
TAEA-PIP0.01	10.2	1000 ppm, 4 bar	92.4	51.3	This work
PVC-g-PDMA	9.3	950 ppm, 4 bar	93.1	≈67.0	[6]
PEI-(C-PES)/PES	10.1	1000 ppm, 2 bar	90.0		[7]
TFC-SDS	7.5	1000 ppm, 6 bar	94.1	47.1	[21]
CCh/PEI-TFC	4.4	1000 ppm, 7 bar	93.0	38.2	[49]
SiO2-PDA/PEI-TFN	5.3	1000 ppm, 6 bar	91.0	≈23.0	[50]
PEI-PEGDGE-PES	3.9	1000 ppm, 4 bar	94.9	46.2	[51]
PDA-PEI/TMC	2.15	1000 ppm, 8 bar	92.4	27.8	[52]
Commercial NF90	10.2	1000 ppm, 6 bar	50.83	60.1	[21]
Commercial NF270	10.9	1000 ppm, 6 bar	50.03	47.8	[21]
